# Rh(III)-catalyzed directed C–H bond amidation of ferrocenes with isocyanates

**DOI:** 10.3762/bjoc.8.212

**Published:** 2012-10-29

**Authors:** Satoshi Takebayashi, Tsubasa Shizuno, Takashi Otani, Takanori Shibata

**Affiliations:** 1 Department of Chemistry and Biochemistry, School of Advanced Science and Engineering, Waseda University, Okubo, Shinjuku, Tokyo, 169-8555, Japan

**Keywords:** amidation, C–H activation, C–H functionalization, ferrocene, rhodium

## Abstract

[RhCp*(OAc)_2_(H_2_O)] [Cp* = pentamethylcyclopentadienyl] catalyzed the C–H bond amidation of ferrocenes possessing directing groups with isocyanates in the presence of 2 equiv/Rh of HBF_4_·OEt_2_. A variety of disubstituted ferrocenes were prepared in high yields, or excellent diastereoselectivities.

## Introduction

Ferrocene and its derivatives are among the most useful organometallic compounds because of their chemical and thermal stabilities, structures, and redox activity [1–2]. One of the most remarkable applications of ferrocene derivatives is as chiral ligands [3–4]. A variety of chiral ferrocenyl ligands with several substitution patterns have been successfully utilized for enantioselective catalysis in both academia and industry. In particular, planar chiral 1,2-disubstituted ferrocenyl scaffolds have been extensively studied, and are among a few premier chiral ligand structures. For instance, a 1,2-disubstituted ferrocenyl ligand, Xyliphos ((*R*)-1-[(*S*)-2-(diphenylphosphanyl)ferrocenyl]ethyl bis(3,5-dimethylphenyl)phosphane) is used for iridium-catalyzed enantioselective hydrogenation to produce the herbicide (*S*)-metolachlor on a scale of more than 10000 tons/year [5].

Planar chiral 1,2-disubstituted ferrocene derivatives are usually synthesized by using diastereoselective *ortho*-lithiation of monosubstituted ferrocenes with an appropriate chiral *ortho*-directing substituent such as chiral amines, sulfoxides, and oxazolines [3]. However, this method suffers from low atom economy, and requires stoichiometric amounts of metal reagents. Functionalization of ferrocene derivatives by transition-metal-catalyzed enantioselective C–H activation is a potentially more atom-economical alternative. However, only a few catalytic C–H activation reactions of ferrocenes have been reported to date, and there is only one report of enantioselective C–H activation of ferrocenes [6–9]. Schmalz et al. reported the first catalytic C–H activation of ferrocenes using a Cu-catalyzed intramolecular carbene insertion into a Cp–H bond [6]. Further, they showed that the reaction could be enantioselective if chiral bisoxazoline ligands were used. However, the substrate scope of this reaction is narrow because of intramolecular reaction. More recently, chiral oxazoline-directed diastereoselective arylation of ferrocenes was reported based on a Pd(II)-catalyzed oxidative coupling reaction [8]. Most of the reactions in this report are, however, stoichiometric, and the yields of the catalytic reactions were low. Although there are a number of reports on stoichiometric directed C–H activation of ferrocenes by using electrophilic metal centers such as Pd(II), Pt(II), and Ru(II) [10–12], this communication describes a metal-catalyzed directed electrophilic C–H activation of an electron-rich Cp ring of ferrocene.

Pentamethylcyclopentadienyl (Cp*)Rh(III) is known to catalyze electrophilic activation of aryl C–H bonds, typically in the presence of an acetate ligand, and is used for oxidative C–C-bond-formation reactions [13]. Recently, several reports of cationic Cp*Rh(III)-catalyzed nonoxidative C–C-bond-formation reactions have been disclosed [14–22]. For example, Ellman et al. and Shi et al. reported that Cp*Rh(III) complexes catalyzed the reaction of aryl C–H bonds to imines, isocyanates, and aldehydes by directed electrophilic activation of aryl C–H bonds at relatively low temperature and under oxidant-free conditions [15–21]. We also reported that cationic Cp*Ir(III) complexes, combined with 1 equiv/Ir of Cu(OAc)_2_, catalyzed the directed C–H activation of aryl C–H bonds at room temperature [23]. In this manuscript, we report application of this nonoxidative Rh(III) catalysis to synthesize planar chiral 1,2-disubstituted ferrocene derivatives.

## Results and Discussion

We chose the reaction of ferrocenyl imine **1a** and phenyl isocyanate as a model reaction and screened several catalysts (Table 1). The cationic Cp*Ir(III) catalyst, which was used in our previous report [23], did not catalyze the reaction at all (Table 1, entry 1). Under copper-salt-free conditions, cationic Cp*Ir(III) catalyst did not give the product, but cationic Cp*Rh(III) selectively afforded monoamidated 1,2-disubstituted ferrocene derivative **2a**, albeit in low yield (Table 1, entries 2 and 3). The catalyst with BF_4_^−^ anion showed the highest activity (Table 1, entries 3–5). The reaction also proceeded in the presence of an isolated cationic Cp*Rh catalyst, which simplifies the reaction setup and is required to prevent the redox reaction between AgBF_4_ and **1a** [24]. However, the use of [RhCp*(MeCN)_3_](BF_4_)_2_ [17] resulted in lower yield likely due to the coordination of MeCN (Table 1, entry 6). The yield significantly increased when the combination of [RhCp*(OAc)_2_(H_2_O)] [25] and 2 equiv/Rh of HBF_4_·OEt_2_ was used to form dicationic Cp*Rh species in situ (Table 1, entry 7) [26].

**Table 1:** Screening of catalysts.

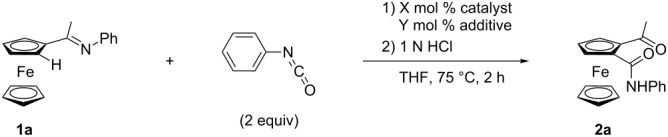			

entry	catalyst (mol %)	additive (mol %)	yield (%)

1^a^	[IrCp*Cl_2_]_2_ (5)	AgSbF_6_ (20), Cu(OAc)·H_2_O (20)	0
2	[IrCp*Cl_2_]_2_ (2.5)	AgSbF_6_ (10)	0
3^b^	[RhCp*Cl_2_]_2_ (2.5)	AgSbF_6_ (10)	30
4^b^	[RhCp*Cl_2_]_2_ (2.5)	AgOTf (10)	20
5^b^	[RhCp*Cl_2_]_2_ (2.5)	AgBF_4_ (10)	42
6	[RhCp*(MeCN)_3_](BF_4_)_2_ (10)	none	29
7	[RhCp*(OAc)_2_(H_2_O)] (10)	HBF_4_·OEt_2_ (20)	86

^a^The reaction was examined at 100 °C for 1 h in 1,2-dichloroethane using 4-methoxyphenyl isocyanate. ^b^The reaction time was 24 h.

We examined a variety of isocyanates under the same reaction conditions given in entry 7 in Table 1 except with a lower catalyst loading of [RhCp*(OAc)_2_(H_2_O)]. The reaction proceeded smoothly with 5 mol % of [RhCp*(OAc)_2_(H_2_O)] along with a slight decrease of yield (Table 2, entry 1). Both electron-rich and -poor aryl isocyantes showed similar reactivity in the present reaction (Table 2, entries 2 and 3). The use of benzyl isocyanate also formed the monoamidated product **2d** (Table 2, entry 4). It required a longer reaction time, but alkyl isocyanates were also available.

**Table 2:** Scope of isocyanates.

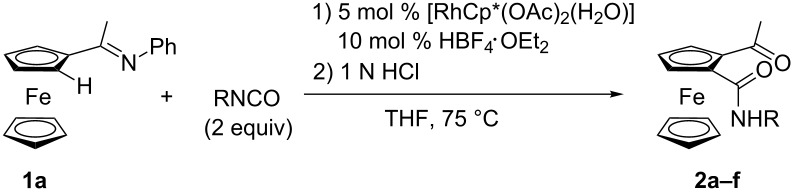				

entry	R	time (h)	product	yield (%)

1	Ph	3	**2a**	74
2	4-MeOC_6_H_4_	3	**2b**	85
3	4-ClC_6_H_4_	3	**2c**	87
4	benzyl	3	**2d**	84
5	*n*-butyl	24	**2e**	41
6	cyclohexyl	24	**2f**	19

**Figure 1:**
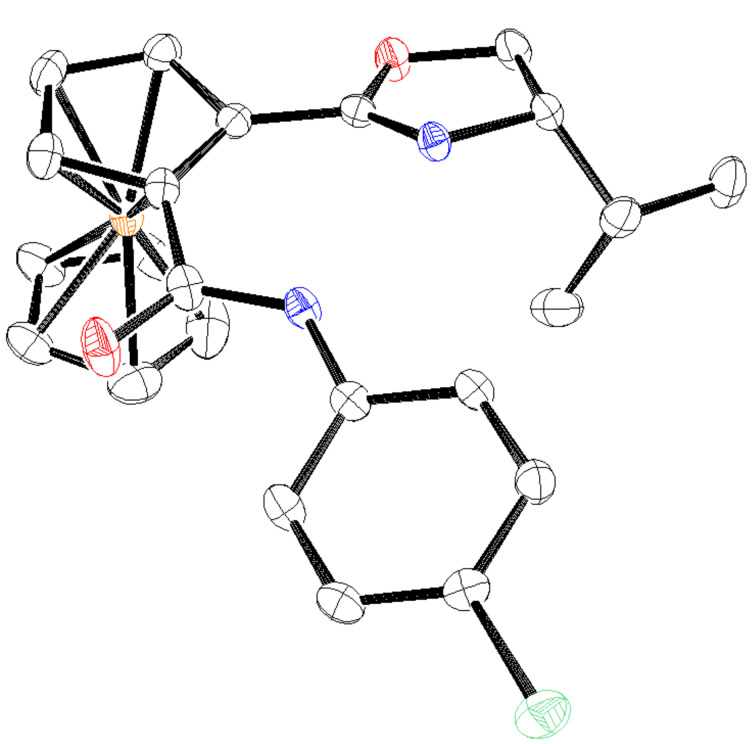
The ORTEP drawing of **3c** with 30% probability ellipsoids, and Flack absolute structure parameter of 0.003(12).

We next examined a diastereoselective reaction using a commercially available chiral oxazolyl ferrocene **1b**, and the reaction was conducted under the same reaction conditions used in Table 2. Several isocyanates were submitted to the reaction with **1b**, and planar chiral 1,2-disubstituted ferrocenes **3a**–**f** were obtained with high diastereoselectivity, but the yields in all cases were moderate because of a low conversion ratio (Table 3). Lower coordination ability of the oxazolyl group compared to the imino one probably decreased the reactivity. The absolute configuration of planar chirality in **3c** was determined to be *S* by X-ray crystallography (Figure 1). The absolute configuration is consistent with the previous report of diastereoselective *ortho*-lithiation of **1b** [27].

**Table 3:** Diastereoselective reaction by using chiral oxazolyl ferrocene **1b**.

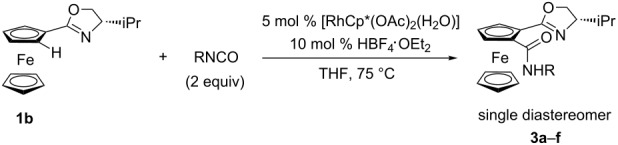				

entry	R	time (h)	product	yield (%)

1	Ph	2	**3a**	43
2	4-MeOC_6_H_4_	2	**3b**	24
3	4-ClC_6_H_4_	2	**3c**	38
4	benzyl	2	**3d**	32
5	*n*-butyl	24	**3e**	69
6	cyclohexyl	24	**3f**	38

## Conclusion

In conclusion, a Cp*Rh(III)-catalyzed reaction between ferrocenyl C–H bonds and isocyanates was developed to synthesize a variety of 1,2-disubstituted ferrocenes. The use of the commercially available chiral oxazolyl ferrocene enabled us to synthesize planar chiral 1,2-disubstituted ferrocenes with excellent diastereoselectivity. The present reaction is a rare example of catalytic methods to construct planar chiral ferrocenes. We are currently investigating an enantioselective reaction.

## Supporting Information

File 1 Experimental procedures and physical properties of new compounds.

File 2 CIF of complex **3c**.
