# Supplementary material for: Rh(III)-catalyzed directed C–H bond amidation of ferrocenes with isocyanates
Source: Beilstein J Org Chem. 2012 Oct 29;8:1844–8. doi: 10.3762/bjoc.8.212 (PMC3511021; doi:10.3762/bjoc.8.212)
Supplement: File 1 — Experimental procedures and physical properties of new compounds. [file Beilstein_J_Org_Chem-08-1844-s001.pdf]

Supporting Information

for

Rh(III)-catalyzed directed C–H bond amidation of ferrocenes with isocyanates

Satoshi Takebayashi, Tsubasa Shizuno, Takashi Otani, and Takanori Shibata*

Address: Department of Chemistry and Biochemistry, School of Advanced Science and Engineering, Waseda University, Okubo, Shinjuku, Tokyo, 169-8555, Japan

Email: Takanori Shibata* - tshibata@waseda.jp

* Corresponding author

Experimental procedures and physical properties of new compounds

Materials and methods. All catalytic reactions were carried out under an argon atmosphere by using oven-dried Schlenk tubes equipped with a magnetic stir bar and a J. Young valve. All solvents were used after degassing with argon. Chloroform-*d* was obtained from Cambridge Isotope Laboratories, and was used as received. THF was dried by using a solvent-drying unit. $[RhCp^{*}Cl_2]_2$ was purchased from Umicore. Ferrocenyl imine **1a** was prepared by following a reported procedure [1]. Common chemicals were purchased from Aldrich, TCI, Strem, Wako Pure Chemical Industries Ltd., and Kanto Chemical Co., and were used as received unless stated otherwise. Flash column chromatography was performed on silica gel (Kanto Chemical Co. 60 N 40–50 μm). Preparative TLC was carried out on silica gel (Merck 60 GF254). NMR spectra were measured on a JEOL ECS400 (400 MHz) spectrometer. ^{1}H and ^{13}C NMR chemical shifts are reported in parts per million (δ) relative to TMS with the residual solvent signal as the internal reference (7.26 and 77.16 ppm for ^{1}H and ^{13}C NMR, respectively). Abbreviations for NMR spectra are s (singlet), d

(doublet), t (triplet), q (quartet), dd (doublet of doublet), dsept (doublet of septet), m (multiplet), and br (broad). IR spectra were recorded on a JASCO FT/IR-4100 spectrophotometer, and are reported in frequency of absorption (cm^{-1}). Low-resolution mass spectra (LRMS) were measured on a JEOL GCMateII by using the FAB method. High-resolution mass spectra (HRMS) were measured on a JEOL JMS-T100 CS by using the ESI method or on a JEOL JMS-SX102A by using the FAB method. Optical rotations were measured on a JASCO DIP-370 polarimeter.

Preparation of [RhCp*(OAc)$_2$(H$_2$O)] [2]. Under argon, [RhCp*Cl$_2$]$_2$ (103.1 mg, 0.167 mmol), AgOAc (137.0 mg, 0.821 mmol), and 7 mL of toluene were placed in a Schlenk tube equipped with a magnetic stir bar. The suspension was stirred for 30 min at 75 °C and [RhCp*Cl$_2$]$_2$ was completely dissolved to form a red solution and a white precipitate of AgCl. The silver salt was filtered by using CH_2Cl_2. The red solution was concentrated to form an orange solid of [RhCp*(OAc)$_2$(H$_2$O)]. Yield: 119.1 mg, 95%.

Typical procedure for the amidation of ferrocene derivatives. [RhCp*(OAc)$_2$(H$_2$O)] (1.9 mg, 0.005 mmol), appropriate ferrocene derivatives **1** (0.10 mmol), and solid isocyanates were placed in an oven-dried Schlenk tube equipped with a magnetic stir bar and a J. Young valve. Air in the tube was then replaced by argon by three vacuum–argon refill cycles. In the case of liquid isocyanates, they were added to the tube using a microliter syringe. THF (0.5 mL) was then added to the tube to form a clear red solution. $HBF_4 \cdot OEt_2$ (1.4 μL, 0.010 mmol) was then added to the tube by using a microliter syringe, and washed down by THF (0.5 mL). The tube was then closed by a J. Young valve, and heated at 75 °C in an oil bath. The reaction mixture was stirred at 75 °C for the reaction time described in Tables 1–3. The reaction mixture was then cooled to room temperature. Five drops of 1 N HCl were then added to the tube to hydrolyze the imine. The mixture was stirred for 30 min at room temperature, concentrated under vacuum, and purified using preparative TLC (20 cm × 20 cm) eluted with a 2:1 mixture of hexanes and ethyl acetate.

1-Acetyl-2-[(phenylamino)carbonyl]ferrocene (2a). Red oil. ^{1}H NMR (399.78 MHz, $CDCl_3$, 24.6 °C): δ 2.61 (3H, s, Me), 4.33 (5H, s, C_5H_5), 4.83 (1H, s, C_5H_3), 4.96 (1H, s, C_5H_3), 5.68 (1H, s, C_5H_3), 7.12 (1H, t, J = 8.2 Hz, aromatic C*H*), 7.38 (2H, t, J = 8.2 Hz, aromatic C*H*), 7.84 (2H, d, J = 8.2 Hz, aromatic C*H*), 12.24 (1H, s, N*H*); ^{13}C{^{1}H} NMR (99.54 MHz, $CDCl_3$, 24.9 °C): δ 28.8 (Me), 72.0 (C_5H_5), 73.7 (C_5H_3), 74.6 (C_5H_3), 78.9 (C_5H_3), 79.3 (C_5H_3), 119.9 (aromatic), 123.5 (aromatic), 128.9 (aromatic), 139.3 (aromatic), 166.5 (*C*ONH), 206.6 (*C*OMe) (one signal was not detected due to overlapping with solvent peak); IR (CH_2Cl_2 cast film): 3009, 1671, 1269, 693, 506 cm^{-1}; HRMS-ESI (m/z): $[M + Na]^+$ calcd for $C_{19}H_{17}FeNNaO_2$, 368.0553; found: 368.0547.

1-Acetyl-2-[[(4-methoxyphenyl)amino]carbonyl]ferrocene (2b). Red oil. ^{1}H NMR (399.78 MHz, $CDCl_3$, 24.6 °C): δ 2.57 (3H, s, Me), 3.80 (3H, s, OMe), 4.29 (5H, s, C_5H_5), 4.78 (1H, s, C_5H_3), 4.91 (1H, s, C_5H_3), 5.63 (1H, s, C_5H_3), 6.89 (2H, d, J = 8.8 Hz, aromatic C*H*), 7.72 (2H, d, J = 8.8 Hz, aromatic C*H*), 12.10 (1H,

s, N*H*); $^{13}C\{^1H\}$ NMR (99.54 MHz, $CDCl_3$, 24.9 °C): δ 28.8 (Me), 55.5 (OMe), 72.0 (C_5H_5), 73.6 (C_5H_3), 74.5 (C_5H_3), 77.2 (C_5H_3), 79.0 (C_5H_3), 79.2 (C_5H_3), 114.1 (aromatic), 121.2 (aromatic), 132.6 (aromatic), 155.8 (aromatic), 166.1 (*C*ONH), 206.6 (*C*OMe); IR (CH_2Cl_2 cast film): 3003, 1662, 1623, 1510, 1241 cm^{-1}; HRMS-ESI (*m/z*): $[M + Na]^+$ calcd for $C_{20}H_{19}FeNNaO_3$, 398.0659; found: 398.0640.

1-Acetyl-2-[[(4-chlorophenyl)amino]carbonyl]ferrocene (2c). Red oil. ^1H NMR (399.78 MHz, $CDCl_3$, 23.7 °C): δ 2.58 (3H, s, Me), 4.30 (5H, s, C_5H_5), 4.81 (1H, s, C_5H_3), 4.94 (1H, d, J = 2.4 Hz, C_5H_3), 5.63 (1H, s, C_5H_3), 7.30 (2H, d, J = 8.8 Hz, aromatic C*H*), 7.75 (2H, d, J = 8.8 Hz, aromatic C*H*), 12.31 (1H, s, N*H*); $^{13}C\{^1H\}$ NMR (99.54 MHz, $CDCl_3$, 23.7 °C): δ 28.9 (Me), 72.1 (C_5H_5), 74.0 (C_5H_3), 74.6 (C_5H_3), 77.5 (C_5H_3), 78.6 (C_5H_3), 79.4 (C_5H_3), 121.1 (aromatic), 128.4 (aromatic), 129.0 (aromatic), 137.9 (aromatic), 166.9 (*C*ONH), 206.9 (*C*OMe); IR (CH_2Cl_2 cast film): 2994, 2363, 1640, 1617, 1492 cm^{-1}; HRMS-ESI (*m/z*): $[M + Na]^+$ calcd for $C_{19}H_{16}ClFeNNaO_2$, 404.0117; found: 404.0102.

1-Acetyl-2-[[(phenylmethyl)amino]carbonyl]ferrocene (2d). Red oil. ^1H NMR (399.78 MHz, $CDCl_3$, 24.7 °C): δ 2.50 (3H, s, Me), 4.19 (5H, s, C_5H_5), 4.40–4.61 (2H, m, Bn), 4.70 (1H, s, C_5H_3), 4.84 (1H, s, C_5H_3), 5.24 (1H, s, C_5H_3), 7.11–7.36 (5H, m, aromatic C*H*), 10.50 (1H, s, N*H*); $^{13}C\{^1H\}$ NMR (99.54 MHz, $CDCl_3$, 25.0 °C): δ 28.8 (Me), 43.6 (Bn), 71.8 (C_5H_5), 73.3 (C_5H_3), 74.8 (C_5H_3), 76.9 (C_5H_3), 78.1 (C_5H_3), 79.0 (C_5H_3), 127.0 (aromatic), 127.7 (aromatic), 128.5 (aromatic), 139.1 (aromatic), 168.4 (*C*ONH), 205.8 (*C*OMe); IR (CH_2Cl_2 cast film): 3320, 1655, 1569, 1411, 697 cm^{-1}; HRMS-ESI (*m/z*): $[M + Na]^+$ calcd for $C_{20}H_{19}FeNNaO_2$, 382.0710; found: 382.0696.

1-Acetyl-2-[(*n*-butylamino)carbonyl]ferrocene (2e). Brownish-red oil. ^1H NMR (399.78 MHz, $CDCl_3$, 26.2 °C): δ 0.98 (3H, d, J = 7.2 Hz, Me Bu), 1.41–1.66 (4H, m, CH_2 Bu), 2.54 (3H, s, CO*Me*), 3.31–3.48 (2H, m, NCH_2), 4.26 (5H, s, C_5H_5), 4.71 (1H, brs, C_5H_3), 4.86 (1H, brs, C_5H_3), 5.56 (1H, brs, C_5H_3), 10.00 (1H, s, N*H*); $^{13}C\{^1H\}$ NMR (99.54 MHz, $CDCl_3$, 26.2 °C): δ 13.9 (Me Bu), 20.3 (CH_2 Bu), 28.9 ($COCH_3$), 31.6 (CH_2 Bu), 39.3 (CH_2 Bu), 71.8 (C_5H_5), 73.1 (C_5H_3), 74.8 (C_5H_3), 76.8 (C_5H_3), 78.7 (C_5H_3), 79.0 (C_5H_3), 168.0 (*C*ONH), 205.8 (*C*OMe); IR (CH_2Cl_2 cast film): 1957, 1655, 1570, 1412, 826 cm^{-1}; HRMS-FAB (*m/z*): $[M + H]^+$ calcd for $C_{17}H_{21}FeNO_2$, 327.0922; found: 327.0920.

1-Acetyl-2-[(cyclohexylamino)carbonyl]ferrocene (2f). Red oil. ^1H NMR (399.78 MHz, $CDCl_3$, 22.0 °C): δ 1.22–1.84 (10H, m, Cy), 2.53 (3H, s, Me), 3.88–3.96 (1H, m, NC*H*),4.24 (5H, s, C_5H_5), 4.70 (1H, t, J = 2.8 Hz, C_5H_3), 4.85 (1H, dd, J = 2.7, 2.8 Hz, C_5H_3), 5.55 (1H, dd, J = 2.7, 2.8 Hz, C_5H_3), 10.03 (1H, s, N*H*); $^{13}C\{^1H\}$ NMR (99.54 MHz, $CDCl_3$, 22.2 °C): δ 24.6 (Cy × 2), 25.8 (Cy), 28.9 (Me), 32.5 (Cy), 33.1 (Cy), 48.0 (Cy), 71.8 (C_5H_5), 73.2 (C_5H_3), 74.8 (C_5H_3), 76.8 (C_5H_3), 79.0 (C_5H_3), 167.0 (*C*ONH), 206.9 (*C*OMe) (one signal was not detected due to overlapping with solvent peak); IR (CH_2Cl_2 cast film): 2929, 1655, 1568, 1412, 1249 cm^{-1}; HRMS-ESI (*m/z*): $[M + Na]^+$ calcd for $C_{19}H_{23}FeNNaO_2$, 374.1022; found: 374.1015.

(*S*)-1-[(4*S*)-4,5-dihydro-4-isopropyl-2-oxazolyl]-2-[(phenylamino)carbonyl]ferrocene (3a). Red oil. ^{1}H NMR (399.78 MHz, $CDCl_3$, 24.6 °C): δ 1.07 (3H, d, *J* = 6.8 Hz, Me), 1.13 (3H, d, *J* = 6.8 Hz, Me), 2.00–2.08 (1H, m, C*H*Me_2), 4.19–4.24 (2H, m, oxazoline), 4.27 (5H, s, C_5H_5), 4.34–4.40 (1H, m, oxazoline), 4.62 (1H, t, *J* = 2.7 Hz, C_5H_3), 4.94 (1H, t, *J* = 2.1 Hz, C_5H_3), 5.46 (1H, t, *J* = 2.2 Hz, C_5H_3), 7.11 (1H, t, *J* = 7.4 Hz, aromatic C*H*), 7.36 (2H, t, *J* = 7.5 Hz, aromatic C*H*), 7.78 (2H, d, *J* = 7.7 Hz, aromatic C*H*), 13.27 (1H, s, N*H*); ^{13}C{^{1}H} NMR (99.54 MHz, $CDCl_3$, 24.8 °C): δ 18.3 (Me), 19.3 (Me), 32.6 (*C*H), 66.4 (C_5H_3), 69.7 (*C*H_2 oxazoline), 71.6 (*C*H oxazoline), 71.8 (C_5H_5), 72.3 (C_5H_3), 74.9 (C_5H_3), 76.7 (C_5H_3), 120.6 (aromatic), 123.7 (aromatic), 129.1 (aromatic), 140.1 (aromatic), 167.5 (*C*ON), 168.3 (*C*ON) (one carbon signal was not detected due to overlapping with solvent peak); IR (CH_2Cl_2 cast film): 2960, 1649, 1317, 1004, 761 cm^{-1}; HRMS-ESI (*m*/*z*): $[M + H]^+$ calcd for $C_{23}H_{25}FeN_2O_2$, 415.1312; found: 415.1310; $[\alpha]_D^{23}$ –123.7 (*c* 0.52, $CHCl_3$).

(*S*)-1-[(4*S*)-4,5-Dihydro-4-isopropyl-2-oxazolyl]-2-[[(4-methoxyphenyl)amino]carbonyl]ferrocene (3b). Red oil. ^{1}H NMR (399.78 MHz, $CDCl_3$, 24.5 °C): δ 1.05 (3H, d, *J* = 6.8 Hz, Me), 1.08 (3H, d, *J* = 6.8 Hz, Me), 1.95–2.03 (1H, m, C*H*Me_2), 3.81 (3H, s, OMe), 4.17–4.23 (2H, m, oxazoline), 4.25 (5H, s, C_5H_5), 4.34–4.40 (1H, m, oxazoline), 4.57 (1H, t, *J* = 2.6 Hz, C_5H_3), 4.91 (1H, dd, *J* = 2.0, 2.6 Hz, C_5H_3), 5.43 (1H, dd, *J* = 2.0, 2.6 Hz, C_5H_3), 6.89 (2H, d, *J* = 9.0 Hz, aromatic C*H*), 7.66 (2H, d, *J* = 9.0 Hz, aromatic C*H*), 13.12 (1H, s, N*H*); ^{13}C{^{1}H} NMR (99.54 MHz, $CDCl_3$, 24.8 °C): δ 18.1 (Me), 18.9 (Me), 32.3 (*C*H), 55.5 (OMe), 66.3 (C_5H_3), 69.5 (*C*H_2 oxazoline), 71.1 (*C*H oxazoline), 71.4 (C_5H_5), 72.0 (C_5H_3), 74.4 (C_5H_3), 76.3 (C_5H_3), 114.0 (aromatic), 121.8 (aromatic), 133.0 (aromatic), 155.7 (aromatic), 167.1 (*C*ON), 168.0 (*C*ON) (one carbon signal was not detected due to overlapping with solvent peak); IR (CH_2Cl_2 cast film): 2963, 1647, 1511, 1242, 749 cm^{-1}; HRMS-ESI (*m*/*z*): $[M + H]^+$ calcd for $C_{24}H_{27}FeN_2O_3$, 445.1418; found: 445.1422; $[\alpha]_D^{23}$ –52.47 (*c* 0.46, $CHCl_3$).

(*S*)-1-[[(4-Chlorophenyl)amino]carbonyl]-2-[(4*S*)-4,5-dihydro-4-isopropyl-2-oxazolyl]ferrocene (3c). Red solid. Mp 177–180 °C; ^{1}H NMR (399.78 MHz, $CDCl_3$, 23.9 °C): δ 1.06 (3H, d, *J* = 6.8 Hz, Me), 1.10 (3H, d, *J* = 6.8 Hz, Me), 1.95–2.07 (1H, m, C*H*Me_2), 4.18–4.24 (2H, m, oxazoline), 4.27 (5H, s, C_5H_5), 4.34–4.43 (1H, m, oxazoline), 4.62 (1H, t, *J* = 2.6 Hz, C_5H_3), 4.95 (1H, t, *J* = 2.2 Hz, C_5H_3), 5.44 (1H, t, *J* = 2.2 Hz, C_5H_3), 7.32 (2H, d, *J* = 8.9 Hz, aromatic C*H*), 7.73 (2H, d, *J* = 8.9 Hz, aromatic C*H*), 13.38 (1H, s, N*H*); ^{13}C{^{1}H} NMR (99.54 MHz, $CDCl_3$, 24.1 °C): δ 18.0 (Me), 19.0 (Me), 32.3 (*C*H), 66.3 (C_5H_3), 69.5 (*C*H_2 oxazoline), 71.4 (*C*H oxazoline), 71.5 (C_5H_5), 71.9 (C_5H_3), 74.7 (C_5H_3), 76.4 (C_5H_3), 121.4 (aromatic), 128.1 (aromatic), 128.7 (aromatic), 138.3 (aromatic), 167.6 (*C*ON), 168.1 (*C*ON) (one carbon signal was not detected due to overlapping with solvent peak); IR (CH_2Cl_2 cast film): 2971, 1646, 1493, 1146, 828 cm^{-1};

HRMS-ESI (*m*/*z*): $[M + H]^+$ calcd for $C_{23}H_{24}ClFeN_2O_2$, 449.0922; found: 449.0916; $[\alpha]_D^{23}$ –98.95 (*c* 0.68, $CHCl_3$).

(*S*)-1-[(4*S*)-4,5-Dihydro-4-isopropyl-2-oxazolyl]-2-[[(phenylmethyl)amino]carbonyl]ferrocene (3d). Red oil. ^1H NMR (399.78 MHz, $CDCl_3$, 23.9 °C): δ 0.86 (3H, d, *J* = 6.8 Hz, Me), 0.88 (3H, d, *J* = 6.8 Hz, Me), 1.66 (1H, dsept, *J* = 5.9, 6.8 Hz, $CHMe_2$), 3.96 (1H, ddd, *J* = 5.9, 8.0, 9.5 Hz, oxazoline), 4.08 (1H, dd, *J* = 8.0, 8.0 Hz, oxazoline), 4.17 (5H, s, C_5H_5), 4.29–4.33 (1H, m, oxazoline), 4.47 (1H, dd, *J* = 5.2, 14.7 Hz, Bn), 4.52 (1H, t, *J* = 2.7 Hz, C_5H_3), 4.72 (1H, dd, *J* = 5.7, 14.7 Hz, Bn), 4.85 (1H, dd, *J* = 1.9, 2.7 Hz, C_5H_3), 5.37 (1H, t, *J* = 1.9 Hz, C_5H_3), 7.24 (1H, t, *J* = 7.5 Hz, aromatic C*H*), 7.32 (2H, t, *J* = 7.5 Hz, aromatic C*H*), 7.40 (2H, d, *J* = 7.5 Hz, aromatic C*H*), 11.74 (1H, brs, N*H*); $^{13}C\{^1H\}$ NMR (99.54 MHz, $CDCl_3$, 24.2 °C): δ 18.4 (Me × 2), 32.3 (*C*H), 43.6 (Bn), 66.7 (C_5H_3), 69.8 (CH_2 oxazoline), 70.9 (*C*H oxazoline), 71.3 (C_5H_5), 72.1 (C_5H_3), 74.3 (C_5H_3), 76.1 (C_5H_3), 76.5 (C_5H_3), 126.9 (aromatic), 127.9 (aromatic), 128.4 (aromatic), 139.6 (aromatic), 167.5 (*C*ON), 168.9 (*C*ON); IR (CH_2Cl_2 cast film): 2960, 1648, 1296, 1007, 749 cm^{-1}; HRMS-ESI (*m*/*z*): $[M + H]^+$ calcd for $C_{24}H_{27}FeN_2O_2$, 429.1469; found: 429.1450; $[\alpha]_D^{23}$ –73.16 (*c* 0.57, $CHCl_3$).

(*S*)-1-[(*n*-Butylamino)carbonyl]-2-[(4*S*)-4,5-dihydro-4-isopropyl-2-oxazolyl]ferrocene (3e). Brownish-red oil. ^1H NMR (399.78 MHz, $CDCl_3$, 26.2 °C): δ 0.96 (3H, d, *J* = 7.4 Hz, Me Bu), 1.04 (3H, d, *J* = 6.8 Hz, Me *i*-Pr), 1.08 (3H, d, *J* = 6.8 Hz, Me *i*-Pr), 1.39–1.48 (2H, m, CH_2 Bu), 1.55–1.65 (2H, m, CH_2 Bu), 1.83–1.91 (1H, m, $CHMe_2$), 3.31–3.51 (2H, m, NCH_2), 4.03–4.15 (2H, m, oxazoline), 4.19 (5H, s, C_5H_5), 4.34 (1H, dd, *J* = 8.4, 9.6 Hz, oxazoline), 4.49 (1H, t, *J* = 2.7 Hz, C_5H_3), 4.84 (1H, t, *J* = 2.3 Hz, C_5H_3), 5.34 (1H, t, *J* = 2.2 Hz, C_5H_3), 11.14 (1H, s, N*H*); $^{13}C\{^1H\}$ NMR (99.54 MHz, $CDCl_3$, 26.7 °C): δ 13.9 (CH_2Me), 18.6 (CH*Me*), 18.7 (CH*Me*), 20.4 (CH_2), 31.9 (*C*H), 32.5 (CH_2), 39.3 (CH_2), 66.6 (C_5H_3), 69.7 (CH_2 oxazoline), 70.7 (*C*H oxazoline), 71.3 (C_5H_5), 72.3 (C_5H_3), 74.2 (C_5H_3), 76.0 (C_5H_3), 167.5 (*C*ON), 168.7 (*C*ON) (one carbon signal was not detected due to overlapping with solvent peak); IR (CH_2Cl_2 cast film): 2959, 1648, 1297, 1180, 749 cm^{-1}; HRMS-FAB (*m*/*z*): $[M + H]^+$ calcd for $C_{21}H_{29}FeN_2O_2$, 397.1579; found: 397.1567; $[\alpha]_D^{33}$ 19.61 (*c* 1.43, $CHCl_3$).

(*S*)-1-[(Cyclohexylamino)carbonyl]-2-[(4*S*)-4,5-dihydro-4-isopropyl-2-oxazolyl]ferrocene (3f). Red oil. ^1H NMR (399.78 MHz, $CDCl_3$, 23.9 °C): δ 1.05 (3H, d, *J* = 6.8 Hz, Me), 1.08 (3H, d, *J* = 6.8 Hz, Me), 1.10–1.50 (10H, m, Cy), 2.05–2.14 (1H, m, $CHMe_2$), 3.91–4.02 (1H, m, Cy), 4.11–4.14 (2H, m, oxazoline), 4.20 (5H, s, C_5H_5), 4.27–4.33 (1H, m, oxazoline), 4.48 (1H, t, *J* = 2.7 Hz, C_5H_3), 4.82 (1H, t, *J* = 2.3 Hz, C_5H_3), 5.34 (1H, t, *J* = 2.2 Hz, C_5H_3), 11.00 (1H, s, N*H*); $^{13}C\{^1H\}$ NMR (99.54 MHz, $CDCl_3$, 24.2 °C): δ 18.1 (Me), 18.9 (Me), 25.2 (Cy), 25.3 (Cy), 25.9 (Cy), 32.0 (*C*H), 32.9 (Cy), 33.8 (Cy), 48.5 (Cy), 66.6 (C_5H_3), 69.1 (CH_2 oxazoline), 70.7 (*C*H oxazoline), 71.4 (C_5H_5), 72.2 (C_5H_3), 74.2 (C_5H_3), 76.1 (C_5H_3), 77.2 (C_5H_3),

167.5 (CON), 167.6 (CON); IR (CH_2Cl_2 cast film): 2926, 1645, 1582, 1449, 1002 cm^{-1}; HRMS-ESI (m/z): $[M + H]^+$ calcd for $C_{23}H_{31}FeN_2O_2$, 421.1782; found: 421.1792; $[\alpha]_D^{23}$ –74.27 (c 0.83, $CHCl_3$).

Crystal structure of 3c

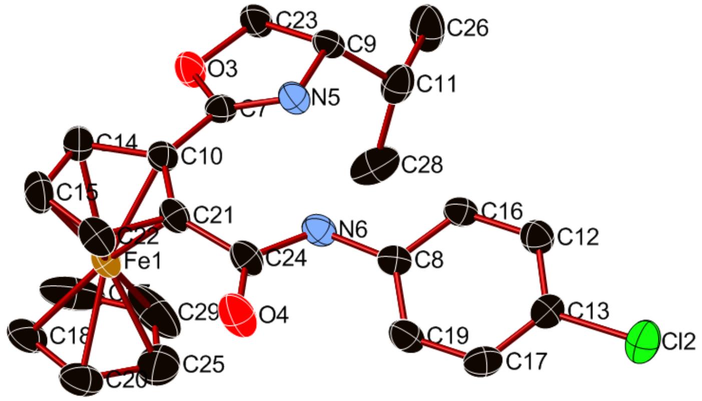

Figure S1: Perspective view of molecule **3c** showing the atom labeling scheme. Non-hydrogen atoms are represented by Gaussian ellipsoids at the 50% probability level.

A. Crystal Data

Empirical Formula	$C_{23}H_{23}ClFeN_2O_2$
Formula Weight	450.75
Crystal Color, Habit	red, block
Crystal Dimensions	0.600 × 0.500 × 0.250 mm
Crystal System	orthorhombic
Lattice Type	Primitive
Lattice Parameters	a = 9.7263(6) Å
	b = 12.2692(7) Å
	c = 17.168(2) Å
	V = 2048.7(3) $Å^3$
Space Group	$P2_12_12_1$(#19)
Z value	4
D_{calc}	1.461 g/cm^3
F_{000}	936.00
μ(MoKα)	8.876 cm^{-1}

B. Intensity Measurements

Diffractometer	R-AXIS RAPID
Radiation	MoKα (λ = 0.71075 Å)
	graphite monochromated
Voltage, Current	50 kV, 40 mA
Temperature	-100.0 °C
Detector Aperture	280 × 256 mm
Data Images	44 exposures
ω oscillation Range (χ=45.0, ϕ=0.0)	130.0 - 190.0°
Exposure Rate	60.0 sec./°
ω oscillation Range (χ=45.0, ϕ=180.0)	0.0 - 160.0°
Exposure Rate	60.0 sec./°
Detector Position	127.40 mm
Pixel Size	0.100 mm
$^{2\theta}$max	54.9°
No. of Reflections Measured	Total: 19307
	Unique: 4664 (R_{int} = 0.0354)
	Friedel pairs: 2014
Corrections	Lorentz-polarization
	Absorption (trans. factors: 0.745 - 0.801)

Data reduction methods

Of the 19307 reflections that were collected, 4664 were unique (R_{int} = 0.0354); equivalent reflections were merged. The linear absorption coefficient, μ, for Mo-Kα radiation is 8.876 cm^{-1}. A numerical absorption correction was applied which resulted in transmission factors ranging from 0.745 to 0.801. The data were corrected for Lorentz and polarization effects.

Structure solution and refinement methods

The structure was solved by direct methods [3] and expanded using Fourier techniques. The non-hydrogen atoms were refined anisotropically. Hydrogen atoms were refined using the riding model. The final cycle of full-matrix least-squares refinement [4] on F^2 was based on 4664 observed reflections and 298 variable

parameters and converged (largest parameter shift was 0.00 times its esd) with unweighted and weighted agreement factors of:

$$R1 = \Sigma ||F_o| - |F_c|| / \Sigma |F_o| = 0.0304$$

$$wR2 = [\Sigma (w (F_o^2 - F_c^2)^2)/ \Sigma w(F_o^2)^2]^{1/2} = 0.0753$$

The standard deviation of an observation of unit weight [5] was 1.03. Unit weights were used. The maximum and minimum peaks on the final difference Fourier map corresponded to 0.46 and -0.20 $e^-/Å^3$, respectively. The absolute structure was deduced based on the Flack parameter, 0.003(12), by using 2014 Friedel pairs [6]. Neutral atom scattering factors were taken from Cromer and Waber [7]. Anomalous dispersion effects were included in F_{calc} [8]; the values for Δf' and Δf" were those of Creagh and McAuley [9]. The values for the mass attenuation coefficients are those of Creagh and Hubbell [10]. All calculations were performed by using the CrystalStructure [11] crystallographic software package except for refinement, which was performed using SHELXL-97 [12].

C. Structure solution and refinement

Structure Solution	Direct Methods (SIR92)
Refinement	Full-matrix least-squares on F^2
Function Minimized	$\Sigma w (F_o^2 – F_c^2)^2$
Least Squares Weights	$w = 1/ [\sigma^2(F_o^2) + (0.0500 \cdot P)^2 + 0.0000 \cdot P]$ where $P = (Max(F_o^2,0) + 2F_c^2)/3$
$2\theta_{max}^{cutoff}$	54.9°
Anomalous Dispersion	All non-hydrogen atoms
No. Observations (All reflections)	4664
No. Variables	298
Reflection/Parameter Ratio	15.65
Residuals: R1 (I>2.00σ(I))	0.0304
Residuals: R (All reflections)	0.0343
Residuals: wR2 (All reflections)	0.0753
Goodness of Fit Indicator	1.027
Flack Parameter (Friedel pairs = 2014)	0.003(12)
Max Shift/Error in Final Cycle	0.004
Maximum peak in Final Diff. Map	0.46 $e^-/Å^3$
Minimum peak in Final Diff. Map	-0.20 $e^-/Å^3$

Table S1: Bond lengths (Å).

atom	atom	distance	atom	atom	distance
Fe1	C10	2.0381(19)	Fe1	C14	2.044(3)
Fe1	C15	2.053(3)	Fe1	C18	2.042(2)
Fe1	C20	2.033(3)	Fe1	C21	2.0368(18)
Fe1	C22	2.042(3)	Fe1	C25	2.037(3)
Fe1	C27	2.040(3)	Fe1	C29	2.030(4)
Cl2	C13	1.748(2)	O3	C7	1.358(3)
O3	C23	1.463(3)	O4	C24	1.223(3)
N5	C7	1.272(3)	N5	C9	1.478(3)
N6	C8	1.403(3)	N6	C24	1.358(3)
C7	C10	1.464(3)	C8	C16	1.394(3)
C8	C19	1.396(3)	C9	C11	1.531(3)
C9	C23	1.533(3)	C10	C14	1.430(3)
C10	C21	1.451(3)	C11	C26	1.532(3)
C11	C28	1.516(4)	C12	C13	1.380(3)
C12	C16	1.387(3)	C13	C17	1.380(3)
C14	C15	1.410(4)	C15	C22	1.417(4)
C17	C19	1.381(3)	C18	C20	1.397(4)
C18	C27	1.413(4)	C20	C25	1.393(4)
C21	C22	1.431(3)	C21	C24	1.496(3)
C25	C29	1.386(6)	C27	C29	1.418(5)

Table S2: Bond angles (°).

atom	atom	atom	angle	atom	atom	atom	angle
C10	Fe1	C14	41.00(8)	C10	Fe1	C15	68.56(8)
C10	Fe1	C18	158.35(10)	C10	Fe1	C20	161.28(9)
C10	Fe1	C21	41.72(8)	C10	Fe1	C22	69.01(8)
C10	Fe1	C25	126.93(10)	C10	Fe1	C27	124.77(10)
C10	Fe1	C29	111.52(11)	C14	Fe1	C15	40.27(10)
C14	Fe1	C18	121.18(11)	C14	Fe1	C20	154.27(10)
C14	Fe1	C21	69.47(9)	C14	Fe1	C22	68.31(10)
C14	Fe1	C25	165.09(11)	C14	Fe1	C27	110.32(12)
C14	Fe1	C29	129.04(13)	C15	Fe1	C18	105.47(11)
C15	Fe1	C20	118.20(10)	C15	Fe1	C21	68.99(9)
C15	Fe1	C22	40.49(9)	C15	Fe1	C25	153.35(12)
C15	Fe1	C27	124.75(13)	C15	Fe1	C29	163.65(13)
C18	Fe1	C20	40.11(11)	C18	Fe1	C21	157.54(9)
C18	Fe1	C22	120.79(10)	C18	Fe1	C25	67.68(12)
C18	Fe1	C27	40.49(11)	C18	Fe1	C29	68.12(12)
C20	Fe1	C21	121.86(9)	C20	Fe1	C22	104.12(10)
C20	Fe1	C25	40.03(12)	C20	Fe1	C27	67.52(11)
C20	Fe1	C29	67.29(13)	C21	Fe1	C22	41.08(8)
C21	Fe1	C25	106.99(11)	C21	Fe1	C27	159.80(11)
C21	Fe1	C29	122.73(11)	C22	Fe1	C25	119.17(11)

Table S2: Bond angles (°) (continued).

atom	atom	atom	angle	atom	atom	atom	angle
C22	Fe1	C27	158.88(12)	C22	Fe1	C29	155.80(13)
C25	Fe1	C27	67.72(14)	C25	Fe1	C29	39.85(15)
C27	Fe1	C29	40.79(15)	C7	O3	C23	105.51(14)
C7	N5	C9	107.87(15)	C8	N6	C24	126.94(16)
O3	C7	N5	118.04(16)	O3	C7	C10	114.94(16)
N5	C7	C10	126.94(17)	N6	C8	C16	117.35(16)
N6	C8	C19	124.04(18)	C16	C8	C19	118.61(18)
N5	C9	C11	111.59(15)	N5	C9	C23	103.49(14)
C11	C9	C23	115.19(16)	Fe1	C10	C7	131.00(13)
Fe1	C10	C14	69.73(12)	Fe1	C10	C21	69.09(10)
C7	C10	C14	121.65(18)	C7	C10	C21	130.44(17)
C14	C10	C21	107.65(17)	C9	C11	C26	109.61(17)
C9	C11	C28	112.47(19)	C26	C11	C28	111.59(19)
C13	C12	C16	119.02(19)	Cl2	C13	C12	119.40(15)
Cl2	C13	C17	119.72(15)	C12	C13	C17	120.87(18)
Fe1	C14	C10	69.27(12)	Fe1	C14	C15	70.19(13)
C10	C14	C15	108.4(2)	Fe1	C15	C14	69.54(13)
Fe1	C15	C22	69.33(13)	C14	C15	C22	108.4(2)
C8	C16	C12	121.10(18)	C13	C17	C19	120.04(18)
Fe1	C18	C20	69.59(13)	Fe1	C18	C27	69.68(15)
C20	C18	C27	107.3(3)	C8	C19	C17	120.32(19)
Fe1	C20	C18	70.30(14)	Fe1	C20	C25	70.14(15)
C18	C20	C25	109.0(3)	Fe1	C21	C10	69.19(11)
Fe1	C21	C22	69.64(12)	Fe1	C21	C24	122.73(13)
C10	C21	C22	106.62(17)	C10	C21	C24	133.66(17)
C22	C21	C24	119.59(18)	Fe1	C22	C15	70.18(13)
Fe1	C22	C21	69.27(12)	C15	C22	C21	108.83(19)
O3	C23	C9	104.76(15)	O4	C24	N6	123.91(19)
O4	C24	C21	119.34(18)	N6	C24	C21	116.75(17)
Fe1	C25	C20	69.84(15)	Fe1	C25	C29	69.79(19)
C20	C25	C29	108.2(3)	Fe1	C27	C18	69.83(15)
Fe1	C27	C29	69.20(19)	C18	C27	C29	107.3(3)
Fe1	C29	C25	70.36(19)	Fe1	C29	C27	70.0(2)
C25	C29	C27	108.2(3)				

References

(1) Takebayashi, S.; Shibata, T. *Organometallics* **2012**, *31*, 4114–4117.

(2) Boyer, P. M.; Roy, C. P.; Bielski, J. M.; Merola, J. S. *Inorg. Chim. Acta* **1996**, *245*, 7-15.

(3) SIR92: Altomare, A.; Cascarano, G.; Giacovazzo, C.; Guagliardi, A.; Burla, M.; Polidori, G.; Camalli, M. *J. Appl. Cryst.* **1994**, *27*, 435.

(4) Flack, H. D. *Acta Cryst.* **1983**, *A39*, 876-881.

(5) Least Squares function minimized (SHELXL97): $\Sigma w(F_o - F_c^2)^2$ where w = Least Squares weights.

(6) Standard deviation of an observation of unit weight: $[\Sigma w(F_o - F_c^2)^2/(N_o - N_v)]^{1/2}$

where: N_o = number of observations, N_v = number of variables

(7) *International Tables for X-ray Crystallography*, Cromer, D. T.; Waber, J. T. The Kynoch Press: Birmingham, England, 1974; Vol. IV, Table 2.2 A.

(8) Ibers, J. A.; Hamilton, W. C. *Acta Crystallogr.* **1964**, *17*, 781.

(9) *International Tables for Crystallography, Creagh*, D. C.; McAuley, W. J., Wilson A. J. C., Ed.; Kluwer Academic Publishers: Boston, 1992; Vol C, Table 4.2.6.8, pp 219–222.

(10) *International Tables for Crystallography, Creagh*, D. C.; McAuley, W. J., Wilson, A. J. C., Ed.; Kluwer Academic Publishers: Boston, 1992; Vol C, Table 4.2.4.3, pp 200–206.

(11) CrystalStructure 4.0: Crystal Structure Analysis Package, Rigaku Corporation (2000–2010). Tokyo Japan, 196-8666.

(12) SHELX97: Sheldrick, G.M. *Acta Crystallogr.* **2008**, *A64*, 112.
